# The catalytic activity of methyltransferase METTL15 is dispensable for its role in mitochondrial ribosome biogenesis

**DOI:** 10.1080/15476286.2024.2369374

**Published:** 2024-06-24

**Authors:** Christian D. Mutti, Lindsey Van Haute, Michal Minczuk

**Affiliations:** a MRC Mitochondrial Biology Unit, University of Cambridge, Cambridge, UK; b Department of Clinical Neurosciences, University of Cambridge, Cambridge, UK

**Keywords:** Mitochondrial ribosome, epitranscriptomics, methyltransferase, chaperone, mitochondria, ribosomal RNA

## Abstract

Ribosomes are large macromolecular complexes composed of both proteins and RNA, that require a plethora of factors and post-transcriptional modifications for their biogenesis. In human mitochondria, the ribosomal RNA is post-transcriptionally modified at ten sites. The N4-methylcytidine (m^4^C) methyltransferase, METTL15, modifies the 12S rRNA of the small subunit at position C1486. The enzyme is essential for mitochondrial protein synthesis and assembly of the mitoribosome small subunit, as shown here and by previous studies. Here, we demonstrate that the m^4^C modification is not required for small subunit biogenesis, indicating that the chaperone-like activity of the METTL15 protein itself is an essential component for mitoribosome biogenesis.

Mammalian mitochondria maintain and express their own genome – mtDNA – that codes for of 13 essential proteins of the oxidative phosphorylation (OXPHOS) machinery and the RNA components necessary for their translation inside the organelle. For this reason, mitochondria contain their own ribosomes (mitoribosomes). Human mitoribosomes comprise the large subunit (mtLSU) containing 52 proteins and a mtDNA-encoded 16S rRNA, whereas the small subunit (mtSSU) contains 30 proteins and a mitochondrially-encoded 12S rRNA. The biogenesis of the mitoribosomal subunits is mediated by numerous GTPases, helicases, anti-association factors, amongst others, to generate the 55S monosome [1,2] Post-transcriptional rRNA modifications and/or RNA modifying enzymes also play important roles in the biogenesis and function of mitoribosomes. In contrast to eukaryotic cytosolic and bacterial rRNAs, which contain over 200 and 30 modifications in rRNA, respectively, human mitochondrial rRNAs (mt-rRNA) contain only ten modified positions. These modifications show a high degree of evolutionary conservation, indicating that although sparse, they are important for the assembly and structure of the mitoribosome and/or relevant for the translation mechanism. Of these ten modified sites, they constitute only three types of modifications: nucleobase methylation, 2’-O-methylation and pseudouridylation. Recent developments in this field have now identified all the enzymes responsible for these modifications [3].

In the human 16S mt-rRNA of mtLSU, three 2’-*O*-ribose methylations Gm2815, Um3039 and Gm3040 (human mtDNA numbering) are catalysed by MRM1, MRM2 and MRM3, respectively [4,5]. MRM2 and MRM3 have been shown to be important for the biogenesis of mtLSU [6,7]. TRMT61B was the first mammalian methyltransferase shown to act on both mt-tRNAs (m^1^A58; universal tRNA position) and mt-rRNA (mtDNA position m^1^A2617 within the 16S rRNA) [8]. Pseudouridine at mtDNA position m.3067T – Ψ3067 – in 16S rRNA is formed by RPUSD4, with depletion of RPSUD4 causing reduces levels of 16S mt-rRNA and mtLSU assembly defect [9,10]. Within human mtSSU, there are five known post-transcriptional modifications of the 12S rRNA (Figure 1a). Two adjacent positions m^6^_2_A1583 and m^6^_2_A1584 (human mtDNA numbering) are methylated by TFB1M, with inactivation of TFB1M leading to mtSSU assembly defect [13]. The m^5^U1076 modification in 16S mt-rRNA is deposited by TRMT2B, an enzyme with dual function that also modifies human tRNAs at the universal position m^5^U54 [14]. Inactivation of the TRMT2B gene leads to accumulation of intermediate pre-SSU particles in different states [6]. The m^5^C1488 modification, found in helix h44 of 16S mt-rRNA and located at the P-site of the mitoribosome, is modified by NSUN4. This enzyme also interacts with MTERF4 and this NSUN4/MTERF4 complex is required for the assembly of the mature mitoribosome [15,16]. It facilitates 16S mt-rRNA methylation by MRM2 by stabilizing the rRNA to expose the site for modification [7]. The modification at mtDNA position 1486 is the only detectable N4-methylcytidine (m^4^C1486) present within the mammalian mitochondria and possibly the cell [17]. S-adenosyl methionine dependent methyltransferase METTL15 was identified as the enzyme introducing m^4^C1486 based on sequence homology to the *E. coli* enzyme RsmH, which catalyses the formation of m^4^C1402 in helix 44 (h44) of SSU 16S rRNA of *E. coli* [17–22].

**Figure 1.**
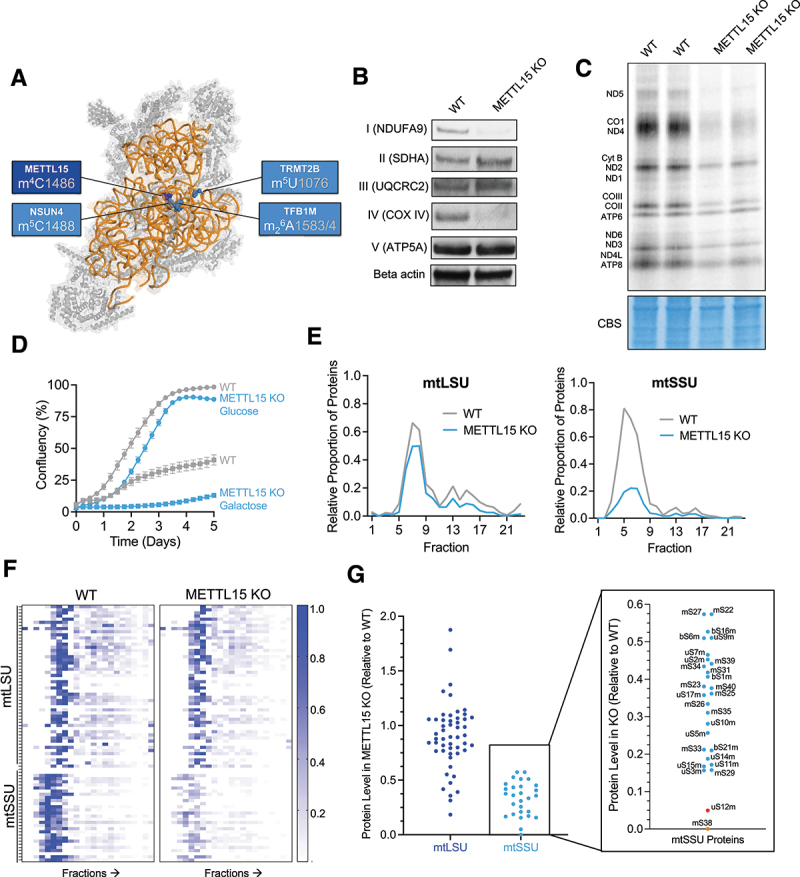
METTL15 is essential for mitochondrial function and small subunit biogenesis.

A, Positions of five modifications of the 12S rRNA of the mtSSU and their respective enzymes are indicated (human mtDNA numbering). Note that these modifications are all in proximity to the decoding centre of the mitoribosome. PDB: 6ZSG. B, Representative western blot of OXPHOS complex proteins in wild type (WT) and METTL15 KO HEK293T cells. C, Mitochondrial *de novo* translation metabolic assay using ^35^S-methionine to label newly synthesized mitochondrial proteins. Coomassie blue stain (CBS) is used for loading. D, Cell proliferation upon knockout of METTL15. Growth curves obtained by Incucyte S3 imaging of WT and METTL15 KO HEK293T cells grown for 5 days in either glucose or galactose-containing medium. E, Quantitative density gradient mass spectrometry (qDGMS) analysis of overall mtLSU and mtSSU proteins in WT and METTL15 KO HEK293T cells. Cells were labelled in either heavy or light labelled growth medium and the different fractions after sucrose gradient sedimentation were analysed by mass spectrometry. F, Representative heatmap from quantitative density gradient mass spectrometry (qDGMS) of SILAC heavy and light labelled wild type and METTL15 KO cells. Reciprocal labelling was performed showing similar heatmap. Analysis was performed using the R package ComPrAn [11,12]. G, Mitochondrial ribosome protein levels in METTL15 KO cells relative to WT. Data from qDGMS and ComPrAn analysis with reciprocal labelling replicates averaged and fractions 6-9 pooled for mtLSU and fractions 4-8 pooled for mtSSU.

Our previous work performed in nearly-haploid HAP1 cells, demonstrated that lack of METTL15 causes defects in mitoribosomal biogenesis, mitochondrial translation and OXPHOS performance. This disruption in mitoribosomal biogenesis was hypothesized to be caused by destabilization of the 12S rRNA folding during the late-stage assembly of the mitoribosome. Further structural data obtained by other groups have demonstrated the interaction of METTL15 with the assembly factor RBFA that facilitates a change in its conformation, thereby guiding the biogenesis of the mitoribosome [6]. Here, we validate the role of METTL15 in mitochondrial function in a new human cellular model and provide evidence that its catalytic function is not essential for mitochondrial ribosome biogenesis.

## METTL15 is essential for mitochondrial function

Inactivation of METTL15 has been shown in several models to impact mitochondrial function and ribosome biogenesis, with the severity substantially varying across these models [17–19,23]. In order to validate the previous work on the essential role of METTL15, we generated a knockout of the human METTL15 gene locus in HEK293T cells using CRISPR/Cas9 guides targeted to exon 3 (the first coding exon) (Supplementary Figure S1). To confirm that the m^4^C1486 modification was absent in the knockout cells, we used targeted RNA bisulphite sequencing (Supplementary Figure S2). To assess the effect on mitochondrial function following ablation of METTL15 in HEK293T cells, we performed a panel of mitochondrial assays. Firstly, we assessed the steady state levels of the OXPHOS complexes by western blotting and showed that NDUFA9 and COXIV levels were almost undetectable, demonstrating an overall reduction in complex I and IV stability, respectively (Figure 1b). Of the five OXPHOS complexes, complexes I and IV are likely the most effected due to them containing the highest number of mtDNA-encoded subunits (7 and 3, respectively). However, we did not detect a decrease in the steady-state levels of UQCRC2 (complex III) in METTL15 KO cells (Figure 1b). This contrasts with our previous findings [17], where we observed a substantial reduction of UQCRC2 in HAP1 cells following METTL15 inactivation. This disparity could be due to metabolic differences between HEK293T and nearly-haploid HAP1 cells. In order to study the effect of METTL15 KO on mitochondrial translation, we performed metabolic labelling with ^35^S radioactively-labelled methionine. Mitochondrial *de novo* protein synthesis was substantially reduced in METTL15 KO cells, but not completely ablated, similar effects to those seen previously [17] (Figure 1c). To further confirm the deficiencies in mitochondrial respiratory chain performance, we grew METTL15 knockout cells in the presence of galactose as the sole carbon source, which forces cells to rely more heavily on mitochondrial ATP production. Under these conditions, METTL15 KO cells showed almost no growth after five days, whereas the WT cells were able to reach almost 50% confluency (Figure 1d). We observed no significant difference in mtDNA copy number between METTL15 KO cells and WT cells (Supplementary Figure S3a). We detected a high variability in the steady-state levels of both 12S and 16S rRNA in cultured cells, with a tendency towards reduced levels in METTL15 KO cells compared to the controls, particularly evident for 12S mt-rRNA (Suplementary Figure S3b). Taken together, these data demonstrate METTL15 causes a severe defect in mitochondrial translation which is unrelated to mtDNA maintenance.

## METTL15 inactivation disrupts small subunit integrity

Given the detrimental effect on mitochondrial function, we investigated whether this is caused by deregulation of mitochondrial ribosome function. Western blotting experiments indicated a trend of reduced steady-state levels of mtSSU proteins upon *METTL15* inactivation (Supplementary Figure 4). To further scrutinize the impact of METTL15 KO on mtSSU integrity with greater precision, we conducted quantitative density gradient fractionation mass spectrometry (qDGMS). This method combines stable isotope labelling with amino acids in cell culture (SILAC) with sucrose gradient sedimentation protein profiling [11,12]. The qDGMS data showed a similar profile of mtLSU across WT and METTL15 KO cells, but a marked reduction in the overall mtSSU proteins in METTL15 KO cells (Figure 1e). This finding is also consistent when analysing individual mitoribosomal proteins on a heatmap which is representative of replicate reciprocal labelling (Figure 1f). To further illustrate this, we pooled the data from fractions related to mtSSU (4–8) and mtLSU (7–9) sedimentation and quantified protein levels in METTL15 KO relative to WT samples (Figure 1g). Whilst all mtSSU proteins were reduced in METTL15 KO compared to WT, two proteins were reduced to a greater extent; uS12m and mS38, with the latter being undetectable in both reciprocal experiments. These two proteins are in close proximity to the METTL15 modification site, suggesting the importance of C1486 in mitoribosome biogenesis and incorporation of these proteins into the mtSSU. Taken together, these data confirm that METTL15 is essential for correct biogenesis of the mitochondrial ribosome small subunit.

## Complementation with METTL15 catalytic mutant rescues phenotype

**Figure 2.**
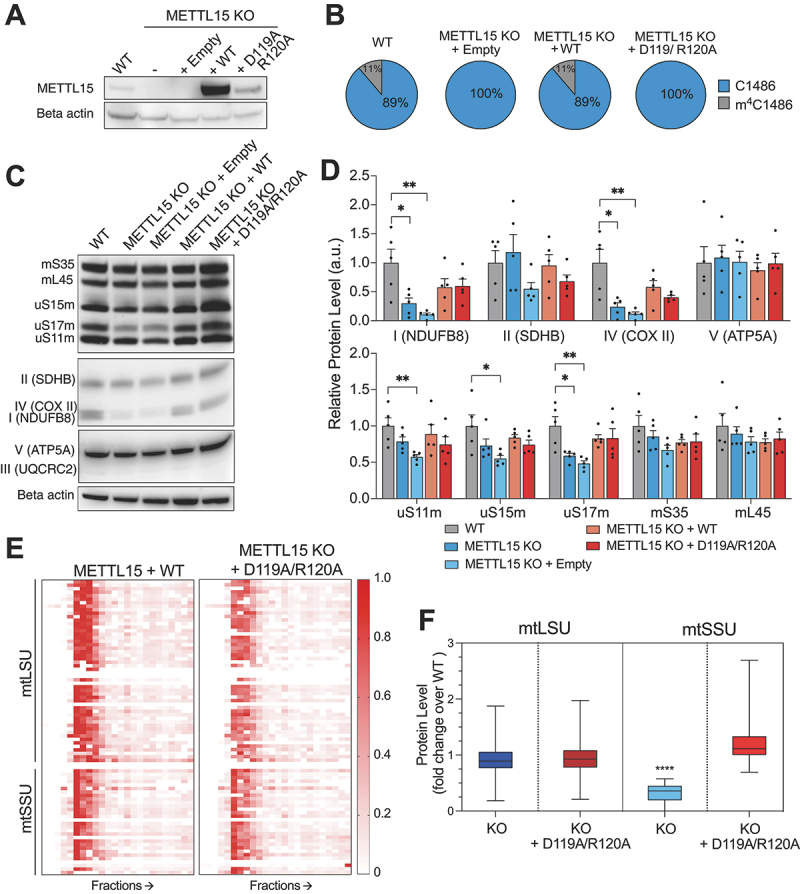
The catalytic activity of METTL15 is not essential for mitochondrial function and small subunit biogenesis. A, Western blot showing complementation of METTL15 KO with WT or catalytic mutant of METTL15. B, Targeted RNA bisulphite sequencing of complemented cells to detect presence of the m^4^C1486 modification. C, Representative western blot showing steady state levels of mitoribosomal and OXPHOS proteins in complemented cell lines. D, Quantification of (c) using.

There are examples of methyltransferase-like proteins in mitochondria, such as METTL17, exhibiting chaperone function rather than enzymatic activity [24]. Thus, our goal was to discern whether the indispensability of METTL15 for mitochondrial function stems from its catalytic activity or its function as an assembly factor. In order to investigate this, METTL15 KO cells were complemented with cDNA encoding for METTL15 that contained amino acid substitutions (D119A and R120A) impairing its ability to bind S-adenosyl methionine and catalysis [17,25]. Cells complemented with an empty plasmid and with WT METTL15 cDNA were used as controls. We confirmed the rescue of METTL15 protein expression by western blotting (Figure 2a). We then used targeted RNA bisulphite sequencing to show comparable m^4^C1486 levels in both WT and KO cells complemented with WT. On contrary, the KO cells complemented with empty or catalytic mutant contained no detectable m^4^C (Figure 2b).

Image J. *n* = 3. Error bars indicate SEM. Statistical analysis was carried out using two tailed Student’s t-test; * *p* < 0.01. **, *p* < 0.001. E, Representative heatmap from quantitative density gradient mass spectrometry (qDGMS) of SILAC heavy and light labelled complemented METTL15 KO cells with wild type METTL15 or catalytic mutant. Analysis was performed using the R package ComPrAn [11,12]. F, Box plot showing average mtLSU and mtSSU protein levels over WT in METTL15 KO cells and following complementation with catalytic mutant. Statistical analysis was carried out using two tailed Student’s t-test; **** *p* < 0.0001.

Next, we aimed to assess the effect of the METTL15 catalytic mutant rescue on mitoribosome and OXPHOS complex protein steady state levels. Our western blotting analysis revealed the most marked effect for the uS17m subunit of mtSSU (Figure 2c-d). Whilst uS17m was significantly reduced in METTL15 KO cells, complementation of both WT and catalytic mutant versions of METTL15 completely rescue its levels (Figure 2d). When assessing the OXPHOS proteins, a similar effect was observed in complexes I and IV (Figure 2c,d). This suggests that the catalytic activity is not essential for the mitoribosome or OXPHOS complex biogenesis. Given that the catalytic mutant can rescue mitochondrial protein steady state levels, we wanted to assess if the catalytic mutant was able to restore mitochondrial function. To this end, we grew cells in glucose or galactose-containing media and measured cell confluency over 8 days (**Supplementary Figure 5a, b**). In these conditions, knockout cells grow slower in both media, whilst the cells complemented with catalytic mutant grow similar to both control cell lines (**Supplementary Figure 5a, b**). Furthermore, mitochondrial translation as measured by ^35^S-Met incorporation was increased following complementation with the catalytic mutant compared with METLL15 KO cells, but is not fully rescued as it is still lower than both WT cells and cells complemented with WT METTL15 (**Supplementary Figure 6**). Given that the METTL15 catalytic mutant is able to rescue mitochondrial function and mtSSU protein steady state levels, we performed complexomics analysis using qDGMS on cells complemented by the catalytic mutant to assess the effect on the mitoribosome more accurately. We showed that mitoribosome subunit levels in cells complemented with catalytic mutant are comparable with KO cells expressing WT METTL15 (Figure 2e), unlike METLL15 KO cells (Figure 2e) and analysis of the individual proteins in fractions referring to mtSSU (4–8), show no difference to control cells (**Supplementary Figure 7**). Altogether, whilst the mtLSU is unaffected by METLL15 KO, the mtSSU is clearly reduced in KO cells, but recovered following the complementation of the D119A R120A catalytic mutant of METTL15 (Figure 2f). These data are inconsistent with the previous report, which was constrained by its reliance solely on steady-state analysis of OXPHOS subunits [17]. This work expands substantially on the previous publication and provides compelling evidence that the methyltransferase activity of METTL15 is not essential for its role in mitochondrial function and that its significance as a chaperone of mtSSU assembly surpasses its catalytic function.

## Discussion

A number of the methyltransferases involved in rRNA modification and ribosome assembly exhibit dual functions, where their enzymatic activity on rRNA is separate from their role in ribosome biogenesis [3]. For example, mammalian NSUN4 has dual function, being involved in 12S rRNA methylation on early stages of mtSSU assembly and facilitating monosome assembly through the interaction with MTERF4, with its methyltransferase activity being dispensable for the later function [15]. Similar findings were reported for MRM2, where the catalytic activity was not important for ribosome function and biogenesis, but the presence of the protein itself was essential [7]. Pet56p, the yeast ortholog of mammalian MRM1, is crucial for mt-LSU maturation regardless of its methyltransferase activity (references within [26]). This also parallels findings for various other proteins involved in bacterial, yeast, and mammalian cytosolic ribosome assembly, such as RsmC, NOP2/NSUN1, or DIMT1 [27–29]. In the case of METTL15, it was initially shown that both its presence and methyltransferase activity are necessary for mitoribosome function, suggesting that the C1486 modification played a role in mitoribosome biogenesis and stability [17–19]. Due to its impact on the assembly of the small subunit and its position within the ribosome, it was speculated that N4 cytidine methylation of position 1486, possibly in combination with m^5^C1488, contributes to the stabilization of 12S rRNA folding, particularly during the late stages of mitoribosomal assembly. However, here we show that the initially suggested secondary role of METTL15 as an assembly factor, as opposed to its methyltransferase activity, is in fact the most important for mitoribosome biogenesis. Complementing METTL15 KO cells with catalytically inactive METTL15 almost completely rescues the mtSSU levels to WT levels. The rationale behind why uS12m and mS38 would be among the proteins with the lowest abundance in the mtSSU-specific fractions in the qDGMS experiments remains unclear if the modification is not deemed necessary. However, it is plausible that the interaction of these proteins with METTL15 could facilitate their incorporation into the late-stage mitoribosome, regardless of the modification.

The observation that cells lacking m^4^C1486 maintain reasonable mitochondrial translation efficiency raises intriguing possibilities. It suggests either (i) the potential redundancy of this modification throughout evolution, with METTL15 protein aiding ribosome biogenesis independently of its catalytic function, or (ii) a role for m^4^C1486 in fine-tuning the structure and function of the decoding centre under specific physiological conditions. The corresponding site in *E. coli* exists as N4, 2′-O-dimethylcytidine (m^4^Cm1402) and interacts with the mRNA’s P-site codon. Without m^4^C1402, non-AUG initiation increases and stop codon read-through decreases [22]. In the mitochondrial ribosome, m^4^C1486 is near the mRNA’s P-site codon, also suggesting a role in decoding. In humans, mitochondrial translation sometimes starts with AUA or AUU instead of AUG. Recent structural study focusing on the mt-rRNA modifications within the mitoribosome, demonstrated that the m^4^C1486 interacts with the phosphate group of the last nucleotide in the P-site mRNA codon via hydrogen bonding [30]. Speculatively, m^4^C1486 methylation may refine the P-site’s structure, impacting on decoding accuracy for these codons, but its importance needs further investigation.

Recent structural studies using cryo-EM have provided further insights into the role of METTL15 in mtSSU assembly [6]. The elucidation of the pre-mtSSU-3 state revealed the folding of 12S rRNA segments h28, h44, and h45, along with the presence of established rRNA modifications. This also enabled visualization of mito-specific RBFA hindering mRNA binding. Concurrently, METTL15 binding and rRNA maturation prompt a significant conformational alteration in RBFA during pre-mtSSU-3 (transitioning from an ‘RBFA-in’ to an ‘RBFA-out’ state), confirming our hypothesis that METTL15 acts as an assembly factor and in line with previous findings that METTL15 co-precipitates with RBFA and a recent structural study confirming their interaction and recognition interface [19,25]. Importantly, the conformational shift is a distinctive trait of human RBFA, unlike its bacterial counterpart. Hence, METTL15 plays a pivotal structural role by guiding the biogenesis pathway and stabilizing a conformation that prevents RBFA’s integration into the mitoribosomal core. The new conformation of RBFA allows the initiation factor mtIF3 to inhabit the subunit interface in advance during the assembly process. Finally, the mitoribosomal protein mS37 displaces RBFA to complete the assembly with the formation of the SSU – mS37–mtIF3 complex that then continues to mtIF2 binding and initiation of translation. These findings were corroborated in a similar study on the assembly of the small subunit of the mitoribosome, where METTL15 is shown to be one of the final assembly factors to be recruited to the late-stage small subunit [31], with that head formation and compaction preceding METTL15 binding and modification. Given the late stage of the ribosome’s assembly, this could explain why the m^4^C modification is not essential as shown here, however, the role of METTL15 as an assembly factor is important as a final checkpoint.

In conclusion, the identification of METTL15 as the methyltransferase responsible for the N4-methylation of cytidine at position 1486 in 12S mt-rRNA sheds light on its role in mitoribosomal biogenesis. The precise molecular function of m^4^C1486, if any, in mRNA decoding requires further investigation. The absence of functional METTL15 affects mitochondrial ribosome assembly, translation, and overall oxidative phosphorylation performance. The methyltransferase activity of METTL15 is not essential for its function in mitochondrial translation, indicating that the protein itself is essential as a factor facilitating mitoribosome biogenesis and/or stability.

## Materials and methods

### Mammalian cell culture

METTL15 was knocked-out in HEK293T using the CRISPR-Cas9 approach targeted to exon 3, followed by screening the clones by PCR and western blotting. Wild-type (WT) and METTL15 KO cells were grown as a monolayer at 37°C in a humidified atmosphere with 5% CO_2_ in high-glucose DMEM (Gibco) supplemented with 10% foetal bovine serum, 100 U/mL penicillin and 100 μg/mL streptomycin.

## Complementation of catalytic mutant

The METTL15 WT cDNA was subjected to site-directed mutagenesis to encode catalytically inactive protein [17]. PCR products were cloned into the pINT-PuroDDEYFP plasmid (Addgene #31442), which along with the pCMVInt (Addgene #18935) integrase plasmid co-transfected (150 ng and 1350 ng, respectively) into METTL15 KO HEK 293T cells for integration into the phiC31 site. For transfections, ~5×10^4^ cells in a 6-well plate were incubated with a mixture of 3.6 μL Lipofectamine (LFA2000), 600 μL Opti-MEM and 1.5 μg of combined plasmids for 4 h at 37°C. 8. Then, 600 μL D-MEM +30% tetracyclin-free serum were added overnight before changing to DMEM containing 100 μg/ml hygromycin and 15 μg/ml blasticidin. The following day the same medium was added, also containing 1 μg/mL puromycin. Approximately 7–10 days following transfection colonies appeared and were screened for the presence of METTL15 protein using western blotting.

## Western blotting

Cell pellets were lysed using lysis buffer (50 mM Tris-HCl pH 7.4, 1 mM EDTA, RNasin Inhibitor, 1% Triton X-100, 1/50 Roche Inhibitor). Lysates were clarified by centrifugation at 10,000 rpm for 5 min at 4°C. The lysates were transferred to clean tubes and kept at −20°C. Protein concentration in clarified lysate was quantified using the PierceTM BCA Protein Assay (ThermoFisher Scientific), following manufacturer’s guidelines. Approximately 30 μg total protein was diluted to an equal volume and combined with NuPAGE LDL Sample Buffer 4× (Invitrogen) and 50 mM DTT. The samples were heated to 92°C for 5 min and loaded on SDS- PAGE 4–12% bis-tris gels (ThermoFisher Scientific) at 200 V for 25 min. Proteins in SDS- PAGE gels were transferred to nitrocellulose membranes using iBlot 2 Dry Blotting System (ThermoFisher Scientific). The membranes were blocked for 1 h with 5% milk in PBST at RT, then primary antibody overnight at 4°C followed by secondary for 1 h at RT. The blots were imaged using an Amersham Imager.

## Metabolic labelling of mitochondrial proteins

In order to label newly synthesized mitochondrially expressed proteins, the previously published protocol was used [32]. Briefly, cells at approximately 80% confluency were incubated in methionine/cysteine-free medium for 10 min before the medium was replaced with methionine/cysteine-free medium containing 10% dialysed FCS and emetine dihydrochloride (100 μg/mL) to inhibit cytosolic translation. Following a 20 min incubation, 120 μCi/ml of [35S]-methionine (Perkin Elmer) was added, and the cells were incubated for 30 min. After washing with PBS, the cells were lysed, and 30 μg of protein was loaded on 10–20% Tris- glycine SDS-PAGE gels. Dried gels were visualized with a PhosphorImager system.

## Cell proliferation

Cell proliferation was measured as previously described [33], Briefly, approximately 80,000 WT and METTL15 KO HEK293T cells were plated per well of a 6 well plate. Cells were grown in either glucose-containing DMEM (4.5 g/L glucose, 110 mg/L sodium pyruvate, 10% FBS, 100 U/mL penicillin, 100 μg/ml streptomycin) or galactose-containing DMEM (0.9 g/L galactose, 110 mg/L sodium pyruvate, 10% FBS, 100 U/mL penicillin, 100 μg/mL streptomycin). Confluency was measured using an Incucyte S3 live-cell imaging system (Essen BioScience). These measurements were taken at 4× zoom, with 9 images per well every 6 hours.

## Mitochondrial DNA copy number analysis

Total cellular DNA was isolated from HEK293T cells using a DNeasy Blood and Tissue kit (Qiagen) according to the manufacturer’s instructions. mtDNA copy numbers were measured by quantitative PCR as previously described [34], using promers provided in Table 1.

**Table 1.** Primers for mtDNA qPCR.

mtDNA	forward	CACCCAAGAACAGGGTTTGT
	reverse	TGGCCATGGGTATGTTGTTAA
	probe	6-Fam-TTACCGGGCTCTGCCATCT-Tamra
B2M	forward	TGCTGTCTCCATGTTTGATGTATCT
	reverse	TCTCTGCTCCCCACCTCTAAGT
	probe	6-Fam-TTGCTCCACAGGTAGCTCTAGGAGG-Tamra

## RNA extraction and RT-qPCR

Measurements of RNA steady-state levels by RT-qPCR was performed as previously described [35]. Briefly, RNA was extracted with TRIzol reagent (Thermo Fisher Scientific) and treated with TURBO DNase (Thermo Fisher Scientific) according to manufacturer’s instructions. 1 μg RNA was reverse transcribed using Omniscript RT kit (Qiagen) with 0.5 μM random hexamers and 0.5 μM oligo dT primers, with cDNA being amplified using primers provided in Table 2.

**Table 2.** Primers for rRNA RT-qPCR.

GAPDH	forward	GAAGGTGAAGGTCGGAGTCAAC
	reverse	CAGAGTTAAAAGCAGCCCTGGT
	probe	6-Fam-GTTTGGTCCGTATTGGGCGCCT-Tamra
16S rRNA	forward	TTTGCAAGGAGAGCCAAAGC
	reverse	AGACGGGTGTGCTCTTTTAGCT
	probe	6-Fam-AGACCCCCGAAACCAGACGAGCTACC-Tamra
12S rRNA	forward	CCCCAGGTTGGTCAATTTC
	reverse	CGGCTTCTATGACTTGGGTTAA
	probe	6-Fam-TGCAGCCACCGCGGTCA-Tamra

## Targeted RNA bisulphite sequencing

Bisulphite conversion of 2 μg DNase treated RNA was performed using the Imprint DNA Modification kit (Sigma-Aldrich). The reaction mixture was incubated for three cycles of 90°C for 5 min and 60°C for 1 h. Following desalting with Micro Bio-spin 6 chromatography columns (Bio-Rad) twice, RNA was desulphonated by adding an equal volume of 1 M Tris (pH 9.0) and incubated for 1 h at 37°C. After ethanol precipitation, reverse transcription was performed with specific primers (Superscript II, Life technologies). After the first stage PCR with overhang primers, excess primers were removed with Ampure XP beads. An 8 cycles second round PCR was performed with indexed primers (Nextera XT), followed by another clean-up with Ampure XP beads. Quality and concentration were assessed with a D1000 Screentape for TapeStation (Agilent Genomics). Libraries were subjected to high-throughput sequencing using the Illumina MiSeq platform. After quality trimming and 3’ end adaptor clipping with Trim_Galore!, reads longer than 20 nt were aligned to a computationally bisulphite-converted human reference genome (GRCh38) with Bismark.

## Quantitative density gradient analysis by mass spectrometry (qDGMS)

Wild type or METTL15 KO HEK293T cells were grown in ‘heavy’, containing^15^N and^13^C- labelled Arg and Lys, or ‘light’ labelled media. Both cell lines were pooled together equally (as assessed by BCA) and mitoribosomal isolation and fractionation was performed as described previously [17]. 50 μL of each fraction was precipitated with 20× volume of ethanol overnight and the precipitants digested with 1:100 (w/w) of trypsin in 50 mM NH_4_HCO_3_ overnight. Liquid chromatography tandem mass spectrometry (LC – MS/MS) analysis of the peptides was carried out using a Thermo Scientific Orbitrap LTQ XL mass spectrometer and a ProxeonEasyLC nanoscale chromatograph. Spectra were analysed by Thermo Proteome Discoverer software which directed firstly protein identification with Matrix Science Mascot software (configured to query the human Uniprot database) and then performed relative quantification of peptide precursors. The custom R package, ComPrAn, was used to separate results for heavy and light-isotope-labelled peptides into control and treatment, and to identify peptides to represent relative protein abundance and create relative abundance profiles across the sucrose gradient [11,12]. In Figures 1g and 2f, the average protein level is determined by averaging fractions 4–7 for the mtSSU and fractions 6–9 for the mtLSU.

## Supplementary Material

KRNB_2024_0038_Supplementary_Info_MAM030524.docx
