# Supplementary material for: The catalytic activity of methyltransferase METTL15 is dispensable for its role in mitochondrial ribosome biogenesis
Source: RNA Biol. 2024 Jun 24;21(1):23–30. doi: 10.1080/15476286.2024.2369374 (PMC11197891; doi:10.1080/15476286.2024.2369374)

**Supplementary data**

**The catalytic activity of methyltransferase METTL15 is dispensable for its role in mitochondrial ribosome biogenesis**

Christian D. Mutti^1^, Lindsey Van Haute^1^, Michal Minczuk^1,2*^

^1^ MRC Mitochondrial Biology Unit, University of Cambridge, CB2 0XY Cambridge, UK

^2^ Department of Clinical Neurosciences, University of Cambridge, CB2 2PY Cambridge, UK


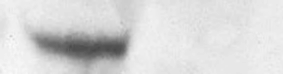


METTL15

Beta actin


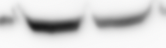


WT

METTL15 KO

**a**

**b**

**Supplementary Figure 1: Generation of HEK293T knockout of *METTL15*.**

**(a)** CRISPR-Cas9 generated knockout of the *METTL15* gene. Guide RNAs were designed to target exon 3. PCR and TOPO cloning used to analyse the METTL15 alleles. Selected clone shows 4 alleles each with varying deletions of exon 3. **(b)** Western blot showing no detection of the METTL15 protein in knockout cell clone, which is detectable in wild-type cells.


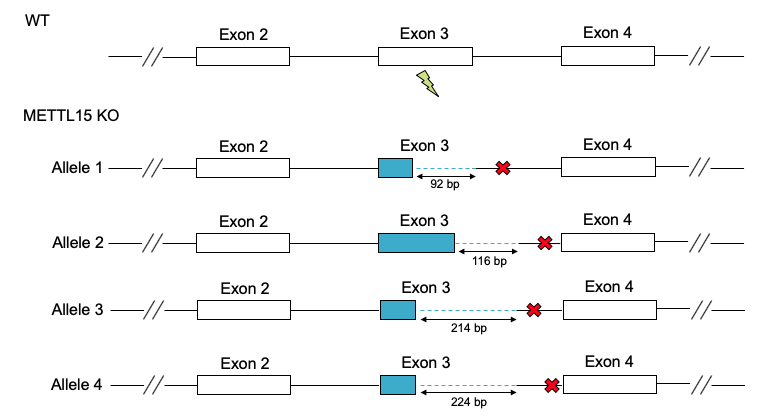

**Supplementary Figure 2: Analysis of m^4^C levels in METTL15 KO.** Targeted RNA bisulfite sequencing of the 12S rRNA in WT and METTL15 knockout cells. Analysis of METTL15 modifying position C1486 and adjacent m^5^C position C1488 modified by NSUN4.

**a**

**b**

**Supplementary Figure 3: Effect of METTL15 inactivation on mtDNA copy number and rRNA levels.** **(a)** mtDNA copy number assessed by qPCR of mtDNA fragments relative to nuclear *B2M* gene in WT and METTL15 KO HEK293T cells. Statistical analysis was carried out using two tailed Student’s t test. n = 3, error bars indicate SEM. **(b)** Reverse transcription qPCR analysis of 12S and 16S rRNA levels in HEK293T cells (WT and METTL15 KO). Steady state levels of mt-rRNA were normalised to GAPDH. Statistical analysis was carried out using two tailed Student’s t-test, no significance was found in any samples. n = 5, error bars indicate SEM.

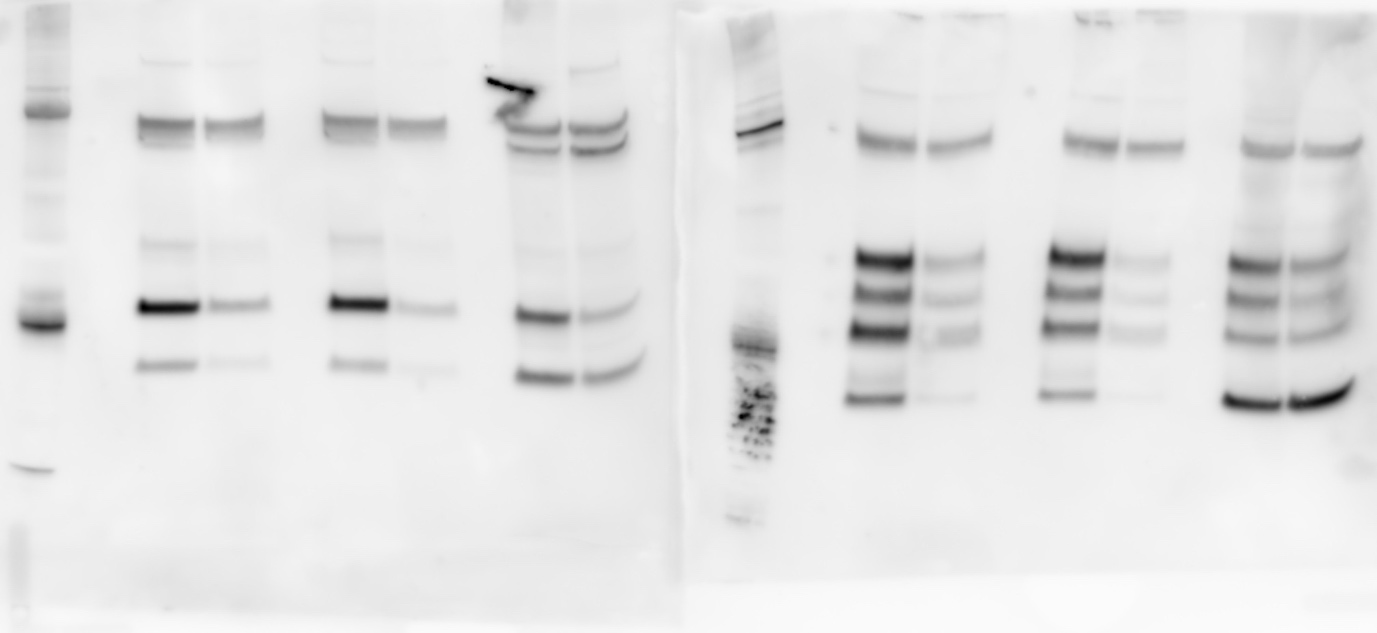

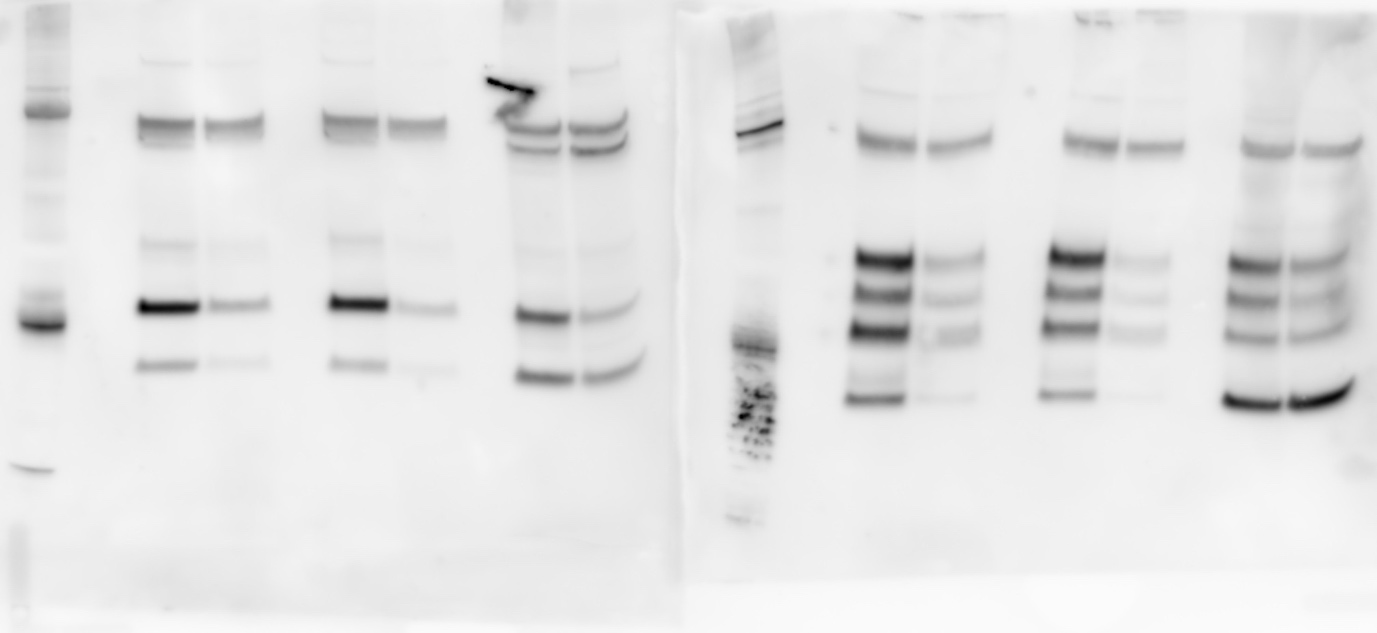

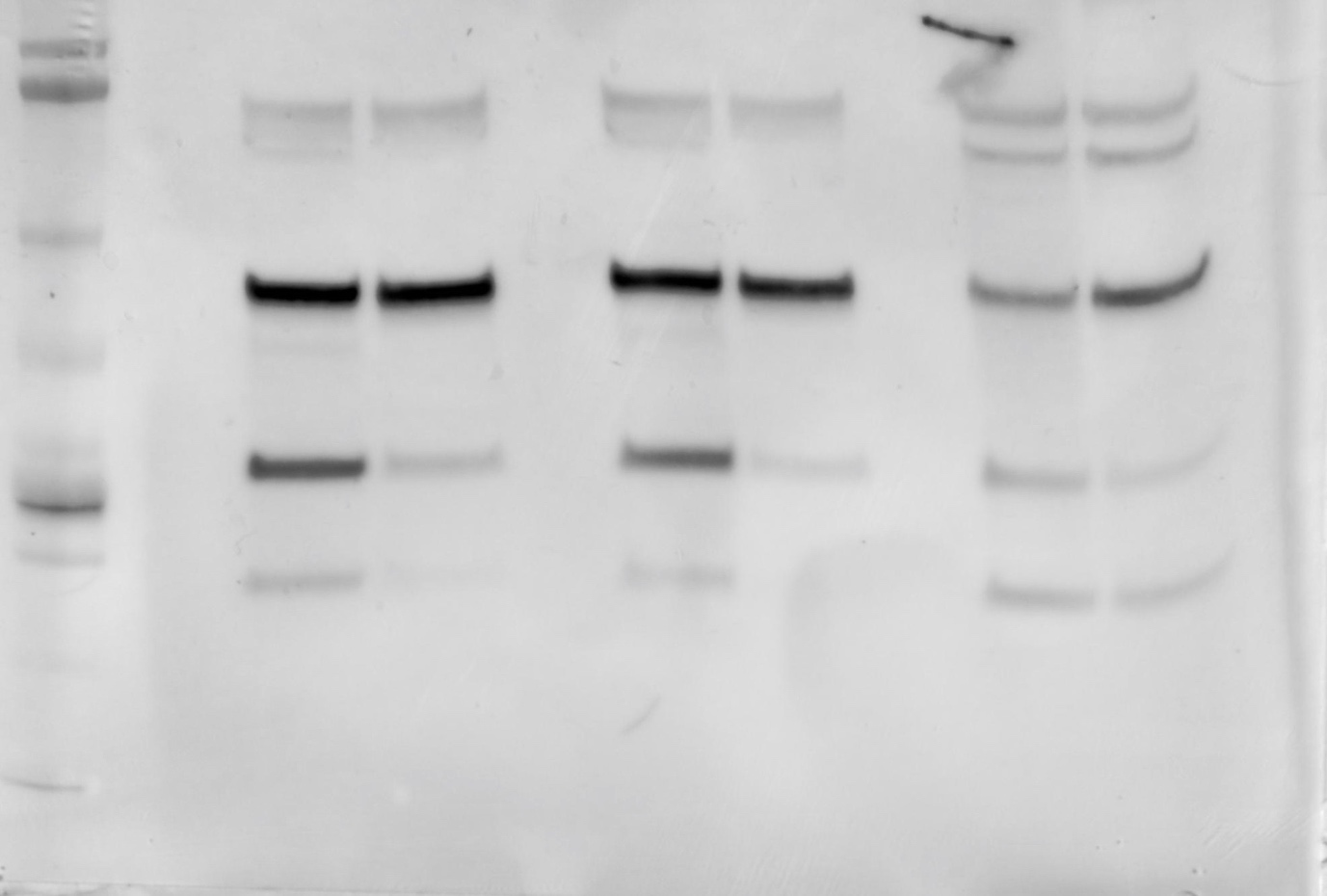


WT KO

uS17m

uS15m

uS11m

mS35

mS40

uL3m

Beta actin

WT KO

WT KO

uL24

**a**

**Supplementary Figure 4: METTL15 inactivation reduces steady-state levels of mitoribosomal small subunit proteins.** **(a)** Western blot analysis of selected mitoribosomal proteins in WT and METTL15 KO HEK293T cells. Beta actin used as a loading control. **(b)** Quantification of (a) using Image J, n = 3. Statistical analysis was carried out using two tailed Student’s t-test. Error bars indicate SEM; * p < 0.01.

**b**

**Supplementary Figure 5: Mitochondrial function in complemented cells. (a, b)** Cell growth assay of complemented METTL15 KO cells. Growth curves obtained by Incucyte S3 imaging of cells grown for 8 days in either glucose- (a) or galactose-containing (b) medium.

**a**

**b**


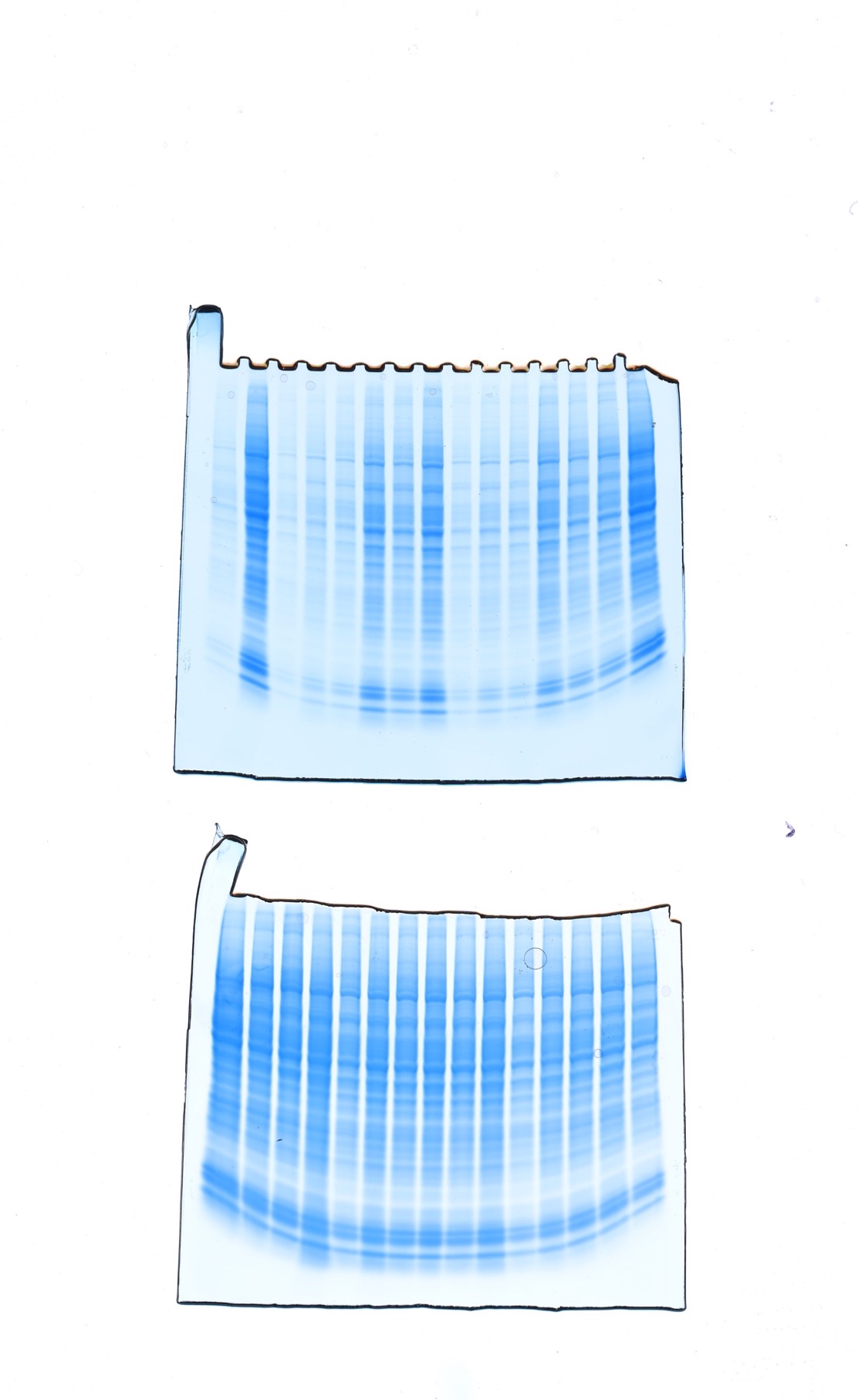

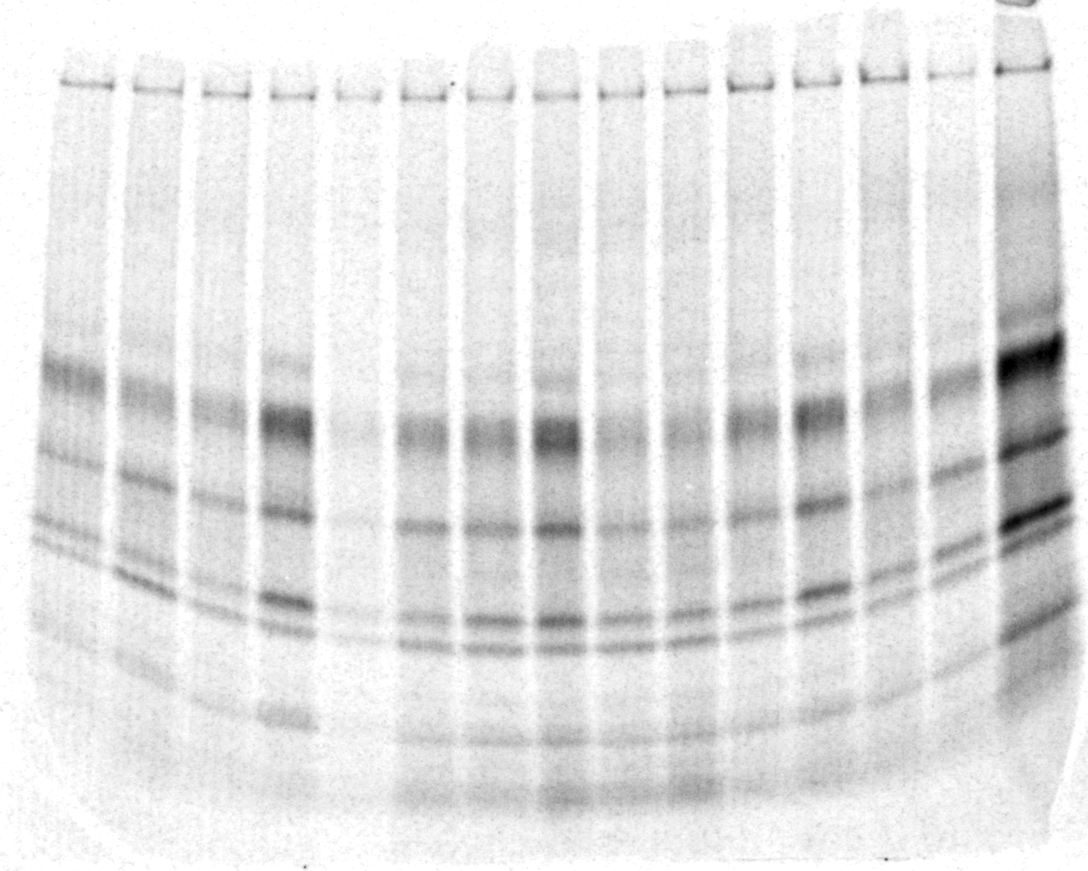


ND5

CO1

ND4

Cyt B

ND2

ND1

COIII

COII

ATP6

ND6

ND3

ND4L

ATP8

CBS

WT

METTL15 KO

KO + EMPTY

KO + WT

KO + D119A R120A

**Supplementary Figure 6: Mitochondrial translation in complemented cells.** Mitochondrial *de novo* translation metabolic assay using ^35^S-methionine to label newly synthesised mitochondrial proteins. Cytosolic translation is blocked using emetine.
^35^S-methionine incubated with cells for 30 mins. Coomassie blue stain (CBS) is used for loading.

**Supplementary Figure 7: Analysis of individual MRP abundance following complementation of METTL15 KO cells.** Mitochondrial ribosome protein levels in METTL15 KO cells complemented wit5h catalytic mutant relative to complemented with WT. Data from qDGMS and ComPrAn analysis with reciprocal labelling replicates averaged and fractions 6-9 pooled for mtLSU and fractions 4-8 pooled for mtSSU.


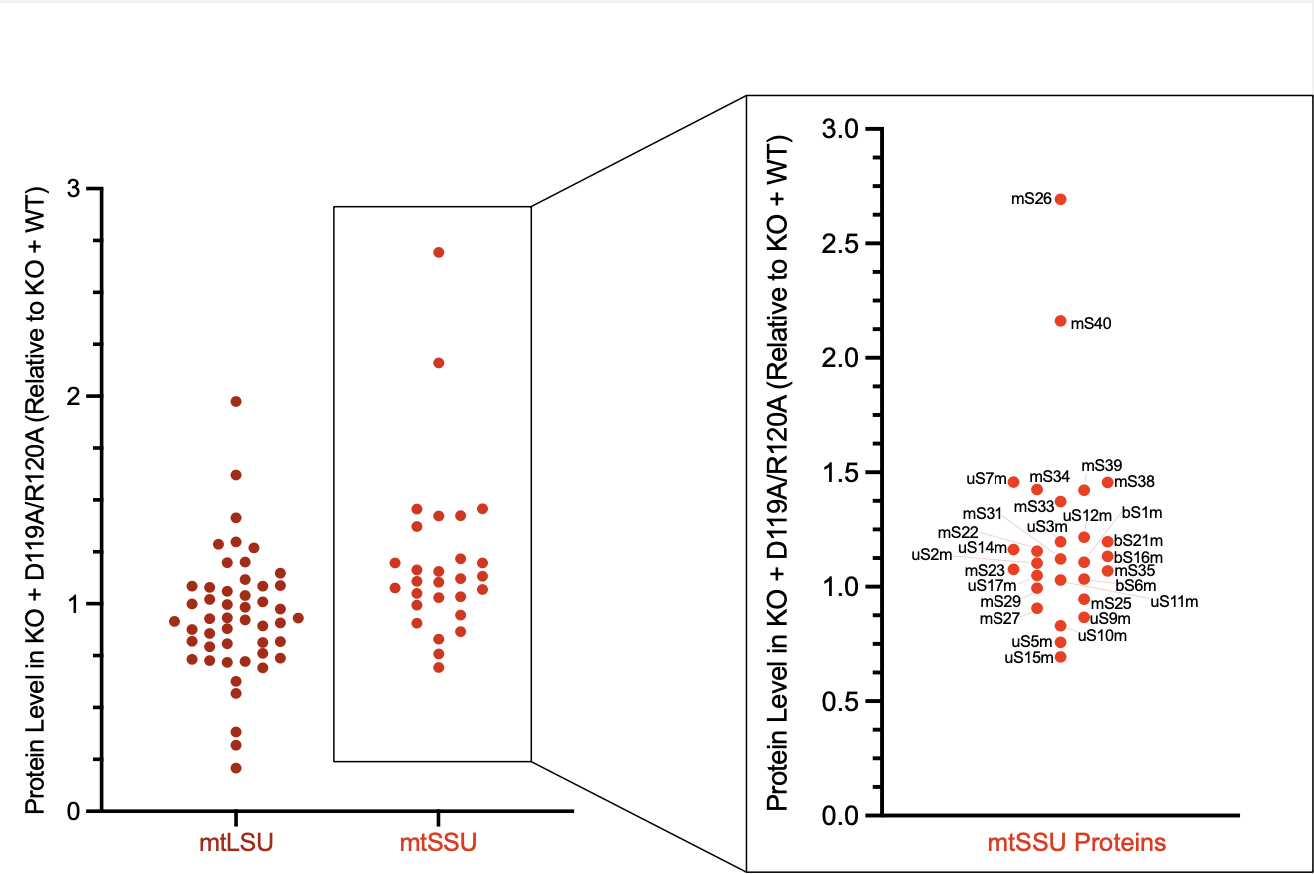

Supplement: KRNB_2024_0038_Supplementary_Info_MAM030524.docx [file KRNB_A_2369374_SM4850.docx]
